# Treatment of a left inguinal hernia with incarceration of the scope during colonoscopy: a case report and literature review

**DOI:** 10.1186/s40792-024-02072-9

**Published:** 2024-11-26

**Authors:** Ryo Numoto, Kohei Taniguchi, Yoshiro Imai, Mitsuhiro Asakuma, Hideki Tomiyama, Shinya Fujiwara, Yoshihiko Nakanishi, Takuya Hamaguchi, Shinsuke Masubuchi, Hitoshi Inoue, Masaru Kawai, Takashi Kinoshita, Shinsho Morita, Michihiro Hayashi, Sang-Woong Lee

**Affiliations:** 1 Department of General and Gastroenterological Surgery, Osaka Medical and Pharmaceutical University, 2-7, Daigaku-Machi, Takatsuki, Osaka 569-8686 Japan; 2 Division of Translational Research, Center for Medical Research & Development, Osaka Medical and Pharmaceutical University, 2-7, Daigaku-Machi, Takatsuki, Osaka 569-8686 Japan; 3 Center for Gastroenterology, Hirakata City Hospital, 2-14-1, Kinyahonmachi, Hirakata, Osaka 573-1013 Japan

**Keywords:** Hernia incarceration, Colonoscopy, Reduction under fluoroscopic guidance

## Abstract

**Background:**

Colonoscopy is widely performed. However, reports of colonoscopic incarceration within inguinal hernias are rare. Incarceration during colonoscopy is a critical condition, and attempting forced reduction may exacerbate complications; therefore, a careful approach is required. Here, we present a case of colonoscopic incarceration of a left inguinal hernia that was successfully reduced under fluoroscopic guidance, followed by elective endoscopic surgery.

**Case presentation:**

A 74-year-old man presented for colonoscopy at a primary care clinic and was referred to our hospital for the incarceration of the colonoscope within the inguinal hernia. On arrival, the colonoscope remained in situ through the anus. Laboratory tests and imaging studies confirmed the absence of perforation. Manual pressure was applied under fluoroscopic guidance to successfully reduce the hernia and allow for scope extraction. No evidence of perforation was revealed in the follow-up fluoroscopic examination using a gastrografin enema. Six weeks later, the patient underwent definitive surgery for total extraperitoneal hernia repair.

**Conclusions:**

A complication of colonoscopy is the incarceration of the colonoscope within the inguinal hernia, particularly in older men. Therefore, inquiring about the patient’s history of inguinal hernia, particularly those accompanied by scrotal swelling, besides assessing the surgical history before performing a colonoscopy, is critical. Furthermore, recent trends include attempts at incarceration reduction under fluoroscopic guidance, with emergency surgery reserved for irreducible cases.

## Background

Despite being a widely performed procedure, colonoscopy can lead to complications such as perforation, bleeding, and occasionally, cardiovascular or pulmonary issues. However, the details of the incarceration of the colonoscope within inguinal hernias are not well known [1]. Such incarceration is a critical complication that may be exacerbated by forced reduction, necessitating a careful approach. In this report, we present the case of a patient with colonoscopic incarceration within a left inguinal hernia, for whom successful reduction was achieved under fluoroscopic guidance, followed by elective endoscopic surgery. Additionally, we discuss the mechanism of colonoscopic incarceration and the use of fluoroscopy-guided techniques, incorporating a review of previously reported literature on this condition to provide a comprehensive understanding of the intraoperative findings.

## Case presentation

**Fig. 1:**
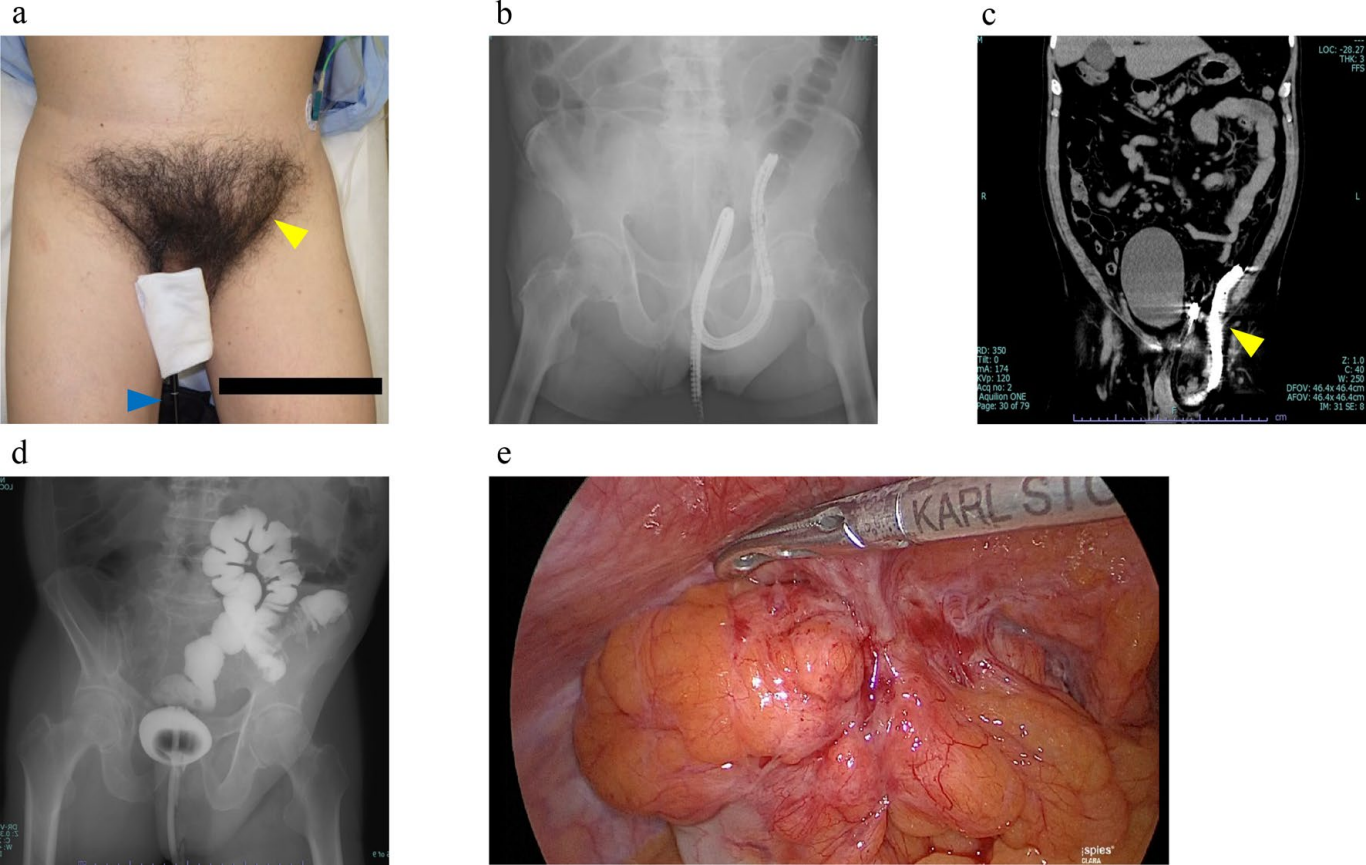
Representative images from abdominal examinations. **a** Colonoscope remained in situ via the anus, and the patient’s left inguinal region was swollen. The blue arrowhead indicates colonoscope remained in situ via the anus. The yellow arrowhead indicates left inguinal bulge. **b** Abdominal radiography showed the colonoscope incarcerated within the left inguinal hernia. **c** Computed tomography scans of the abdomen revealed the presence of the colonoscope. The yellow arrowhead indicates colonoscope within the left inguinal hernia. **d** Fluoroscopic examination using a gastrografin enema showed no signs of perforation. **e** Intraoperative image showing a large direct inguinal hernia, incarceration of the sigmoid colon within the hernia sac, and extensive adhesions around the hernia site

A 74-year-old man presented to a primary care clinic for a routine colonoscopy. During the procedure, the sigmoid colon and the colonoscope were incarcerated in the left inguinal hernia. The patient was referred to our hospital because of difficulties in incarceration reduction. His medical history included hypertension, hyperlipidemia, and type 2 diabetes mellitus; however, no previous surgery had been performed. Upon arrival, his vital signs were stable, except for blood pressure (192/92 mmHg), and the colonoscope remained in situ via the anus. The patient exhibited swelling and tenderness in the left inguinal region and a palpable hard mass that resembled the scope (Fig. 1a). Laboratory tests indicated mildly elevated levels of inflammatory markers (white blood cell 9980/mm^3^, C-reactive protein level: 0.16 mg/dL) without a significant increase in enzymes such as creatine kinase (84 U/L) or lactate dehydrogenase (200 U/L). Abdominal radiography revealed incarceration of the scope within the left inguinal hernia (Fig. 1b). Computed tomography (CT) confirmed the presence of scope and excluded perforation or strangulation, as indicated by the absence of free air or ascites (Fig. 1c). In collaboration with the internal medicine team, after confirming the absence of loop formation, manual pressure was applied (not the pulley technique) under fluoroscopic guidance to reduce the size of the hernia and extract the scope. A follow-up fluoroscopic examination using a gastrografin enema revealed no evidence of perforation (Fig. 1d). The patient resumed oral intake the following day and was discharged on day 3 of hospitalization without any complications. Six weeks later, considering his personal circumstances, he underwent definitive surgery for total extraperitoneal hernia repair.

During surgery, a large direct inguinal hernial defect was observed. Incarceration of the sigmoid colon within the hernia sac and extensive adhesions around the hernia defect were observed (Fig. 1e). Reducing the hernia with an additional 5 mm ports on both sides of the abdomen was challenging because of strong adhesions. Considering the risk of intestinal injury, we opted to perform total extraperitoneal hernia repair. Despite the challenges of large hernia defects and strong adhesions, the extraperitoneal surgery was successful, with no damage to the peritoneum. A Bard 3D Max Light mesh, size L (Medicon, C. R. Bard, Inc.) and an Absorba Tack 5 mm (Nippon Covidien, Ltd., Tokyo, Japan) were used to secure the mesh with three tacks for extraperitoneal surgery. After reinspection of the abdominal cavity to confirm proper mesh placement, the surgery was concluded. The operative time was 179 min, with minimal blood loss. The patient recovered well and was discharged on postoperative day 2 without any recurrence or complications. One month after surgery, no lesions were revealed by total colonoscopy revealed.

## Discussion

Here, we describe a case of colonoscopic incarceration of an inguinal hernia managed manually under fluoroscopic guidance. Our findings demonstrate that emergency surgery can be avoided, and beneficial treatment can be provided in such cases.

Although colonoscopy is commonly performed, the colonoscopic incarceration of an inguinal hernia is rare. Since the first report by Leisser in 1990, only 24 cases have been reported, including ours (Table 1) [2–21]. All reported cases involved older male patients, and 23 of the 24 patients had hernias on the left side. We speculated that the reason all cases involved men was the significantly higher risk of hernia development in them due to anatomical differences. In men, the inguinal canal is slightly wider due to the passage of the spermatic cord, resulting in a hernia risk approximately 9 times higher than in women. Furthermore, the risk increases with age as supportive tissues weaken, leading to enlargement of the hernia opening [22]. The reason for this left-sided predominance is speculated to be the pressure exerted by the insertion and withdrawal of the colonoscope. One patient with right-sided incarceration was diagnosed after a right hemicolectomy for diverticular bleeding, suggesting that surgery-induced anatomical changes or adhesions may affect bowel positioning [4]. In addition, more than half of the patients had undiagnosed hernias before the colonoscopy. Inguinal hernias are common among older men, with a prevalence of 6% [23]; however, without patient-reported symptoms, detection before colonoscopy can be challenging. In our patient, if an inguinal hernia had been detected before the colonoscopy, incarceration of the colonoscope in the hernia and subsequent procedural interruption could have been prevented. Although the patient’s surgical history is crucial, verifying the presence of a hernia before colonoscopy is vital. Furthermore, previous reports have indicated relative contraindications for colonoscopy in the presence of large inguinal hernias, necessitating a discussion of the associated risks [4]. Computed tomographic colonography is the recommended alternative when colonoscopy is necessary prior to hernia repair. When colonoscopy is essential, such as when a malignancy is suspected, the risk of incarceration is reduced by manually reducing the hernia and maintaining bowel straightening during colonoscopy [14]. In our case, colonoscopy was performed after hernia repair because the tumor was not suspected.

**Table 1:** Summary of reports on colonoscopic incarceration of inguinal hernias

Case number	Author (year)	Sex	Age (years)	Hernia history	Side	Situation	Fluoroscopy guidance	Repair method	Contents
Case 1	Leisser et al. (1990) [2]	Male	50	NA	Left	Insertion	‒	Reduction	S colon
Case 2	Fulp et al. (1990) [3]	Male	71	Known	Left	Insertion	−	Reduction	S colon
Case 3	Koltun et al. (1991) [4]	Male	76	Known	Right	Withdrawal	+	Pulley technique	S colon
Case 4	Yamamoto et al. (1994) [5]	Male	83	Known	Left	Insertion	+	Reduction	S colon
Case 5	Saunders (1995) [6]	Male	73	Unknown	Left	Insertion	−	Manual reduction	S colon
Case 6	Punnam et al. (2003) [7]	Male	77	Known	Left	Withdrawal	−	Emergency surgery	S colon
Case 7	Lee et al. (2004) [8]	Male	70	Unknown	Left	Insertion	−	Manual reduction	S colon
Case 8	Iser et al. (2005) [9]	Male	NA	NA	Left	Insertion	−	Manual reduction	S colon
Case 9	Fan et al. (2007) [10]	Male	73	NA	Left	Withdrawal	+	Manual reduction	S colon
Case 10	Kume et al. (2009) [11]	Male	81	Unknown	Left	Withdrawal	+	Manual reduction	S colon + bowel
Case 11	Kubo et al. (2012) [12]	Male	77	Unknown	Left	Insertion	+	Emergency surgery	S colon
Case 12	Tanishima et al. (2012) [13]	Male	83	Known	Left	Insertion	+	Manual reduction	S colon
Case 13	Tan et al. (2013) [14]	Male	76	Unknown	Left	Withdrawal	+	Manual reduction	S colon
Case 14	Kamezaki et al. (2015) [15]	Male	87	Unknown	Left	Insertion	−	Reduction	S colon
Case 15	Tas et al. (2015) [16]	Male	70	Unknown	Left	Withdrawal	+	Emergency surgery	S colon
Case 16	Wang et al. (2017) [17]	Male	70	Known	Left	Insertion	+	Emergency surgery	S colon
Case 17	Ito et al. (2017) [18]	Male	79	Unknown	Left	Insertion	−	Reduction	S colon
		Male	62	Unknown	Left	Insertion	−	Manual reduction	S colon
		Male	73	Known	Left	Insertion	+	Reduction	S colon
Case 18	Kimura et al. (2018) [19]	Male	75	Unknown	Left	Insertion	+	Emergency surgery	S colon
Case 19	Torrealba et al. (2021) [20]	Male	54	Unknown	Left	Insertion	+	Pulley technique	S colon
Case 20	Abe et al. (2022) [21]	Male	73	Unknown	Left	Withdrawal	+	Manual reduction	S colon
		Male	74	Known	Left	Insertion	+	Reduction	S colon
Case 21	Our case (2024)	Male	74	Unknown	Left	Insertion	+	Manual reduction	S colon

*NA* not available, *S* sigmoid

**Fig. 2:**
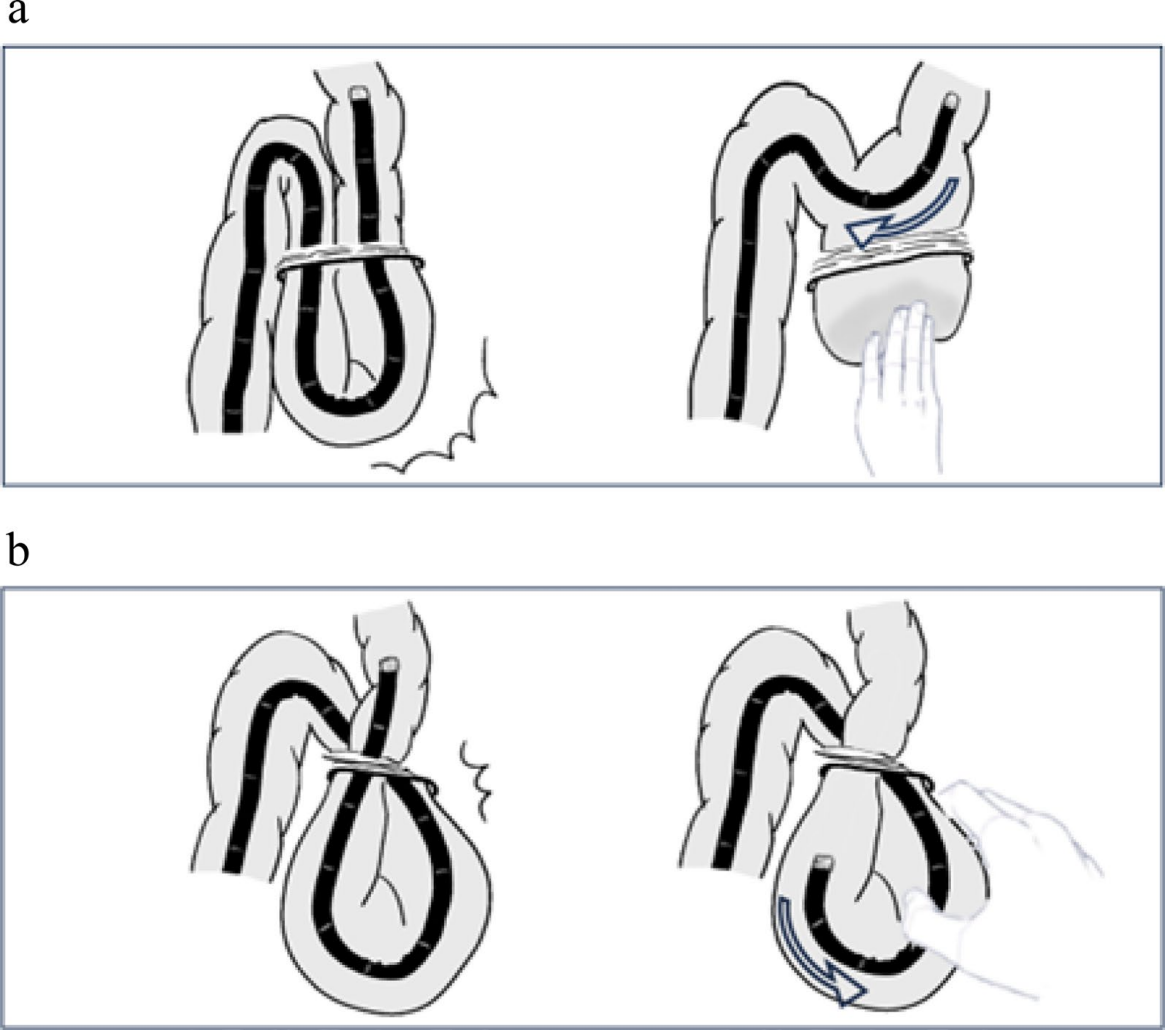
Mechanism of colonoscopy incarceration and reduction techniques. **a** No loop formation. Straightening the colonoscope with manual pressure can facilitate reduction. **b** Loop formation. The pulley technique, wherein the loop arc is grasped when the colonoscope is withdrawn, is effective

Reduction under fluoroscopic guidance has become increasingly common, and cases of emergency surgery have been reported due to extraction difficulties [7, 12, 16, 17, 19]. An effective reduction technique must be devised to manage colonoscopic incarceration based on a thorough understanding of its mechanism. To date, incarceration has occurred either during insertion (*n* = 17, including the present case) or withdrawal (*n* = 7) of the colonoscope. The size of the hernial orifice and the extent of adhesion can influence incarceration, which can lead to entrapment of the bowel and mesentery. During withdrawal, the colonoscope may form a loop originating from the hernia orifice, suggesting potential entrapment. If loop formation does not occur, manual straightening of the colonoscope can facilitate the reduction. However, when loop formation occurs, forced reduction may lead to perforation because the colonoscope and bowel may become trapped in the arcuate portion. In such cases, the pulley technique, which involves grasping the loop arc while withdrawing the colonoscope, has proven to be effective (Fig. 2a, b) [4]. In our patient, the absence of loop formation confirmed under fluoroscopic guidance allowed for successful reduction using manual pressure, and follow-up contrast enema showed no signs of perforation. We believe that by effectively utilizing imaging studies without forced reduction, emergency surgery can be avoided and definitive endoscopic surgery performed.

Although surgery depends on the patient's preference, definitive surgery is often performed 2–3 months after successful manual reduction, and we followed the same approach in this case. Intraoperative findings revealed a left direct inguinal hernia with the sigmoid colon incarcerated within the hernia sac. Additionally, extensive sheet-like adhesions around the hernia orifice were observed, which were likely formed due to repeated prolapse over time. Based on those findings, we determined that the intraperitoneal surgery would be difficult, and opted for the extraperitoneal approach. Our strategy suggest that a total extraperitoneal hernia repair can be safely performed without damaging the intestinal tract.

## Conclusions

Inguinal hernial incarceration is a rare complication of colonoscopy, particularly in older men. Therefore, inquiring about the patient’s inguinal hernia and surgical history before performing a colonoscopy is critical. If the colonoscope becomes incarcerated, the procedure should be paused to evaluate symptoms, such as inguinal pain, and check for serious complications, such as perforation or strangulation. Based on our experience, confirming the absence of perforation through imaging followed by manual reduction under fluoroscopic guidance and elective surgery is an effective strategy for managing this complication.

## Data Availability

All data supporting the conclusions of this study are included in this published article.
